# Immunoregulatory CD4^-^CD8^-^ T cells as a potential therapeutic tool for transplantation, autoimmunity, and cancer

**DOI:** 10.3389/fimmu.2013.00006

**Published:** 2013-01-24

**Authors:** Erin E. Hillhouse, Jean-Sébastien Delisle, Sylvie Lesage

**Affiliations:** ^1^Department of Microbiology and Immunology, University of MontrealMontreal, QC, Canada; ^2^Research Center, Maisonneuve-Rosemont HospitalMontreal, QC, Canada; ^3^Department of Medicine, University of MontrealMontreal, QC, Canada; ^4^Division of Hematology and Oncology, Maisonneuve-Rosemont HospitalMontreal, QC, Canada

**Keywords:** graft-vs-host disease, autoimmunity, cancer, graft tolerance, immunoregulation, double negative T cells

## Abstract

A central objective in organ transplantation and the treatment or prevention of autoimmune disease is the achievement of antigen-specific immune tolerance. An additional challenge in bone marrow transplantation for the treatment of hematological malignancies is the prevention of graft-vs-host disease (GVHD) while maintaining graft-vs-tumor activity. Interestingly, CD4^-^CD8^-^ (double negative, DN) T cells, which exhibit a unique antigen-specific immunoregulatory potential, appear to exhibit all of the properties to respond to these challenges. Herein, we review the therapeutic potential of immunoregulatory DN T cells in various immunopathological settings, including graft tolerance, GVHD, cancer, and autoimmunity.

## INTRODUCTION

CD4^-^CD8^-^ double negative (DN) T cells compose approximately 1–3% of total T cells in both mice and humans (Strober et al., 1989; Abraham et al., 1992; Fischer et al., 2005). Phenotypically, this rare T cell subset expresses a polyclonal αβT cell receptor (TCR) repertoire, lacks the expression of Foxp3 as well as natural killer (NK) cell markers and mostly presents with a naïve T cell phenotype (recently reviewed; Hillhouse and Lesage, 2012). Interestingly, their distinct phenotype, namely the lack of CD4 and CD8 co-receptors, is also believed to influence the method by which DN T cells recognize antigens and subsequently signal through their TCR. In fact, major histocompatibility complex (MHC) restriction is the cardinal feature of antigen recognition by T cells, where the CD4 and CD8 co-receptors respectively facilitate the interaction with MHC class II and I molecules. As DN T cells lack both CD4 and CD8 co-receptors, the means by which the αβTCR on the DN T cell recognizes MHC ligands with sufficient affinity and avidity to provide activation of DN T cells is unclear. It has been recently proposed that DN T cells recognize non-MHC ligands. Indeed, using quad-deficient mice that lack the expression of CD4 and CD8 co-receptors as well as MHC class I and II expression, Van Laethem et al. (2007) demonstrated that T cell development could proceed in an MHC-independent manner. In fact, in the absence of CD4 or CD8 co-receptor expression, the intracellular tyrosine kinase Lck is no longer sequestered by the co-receptors and is thus available to promote MHC-independent TCR signaling in thymocytes. Subsequently, these thymocytes develop into mature DN T cells that enter the periphery (Van Laethem et al., 2007). The ability of DN T cells to recognize non-MHC ligands is also supported by the finding that mature DN T cells from quad-deficient mice proliferate vigorously against both MHC-sufficient and MHC-deficient stimulator cells, but not in the absence of stimulator cells (Van Laethem et al., 2007; Tikhonova et al., 2012). In fact, a recent study has revealed the native self-protein CD155 as at least one of the ligands recognized by quad-deficient DN T cells via their TCR and in the absence of antigen-processing (Tikhonova et al., 2012). Therefore, the quad-deficient mouse model has helped to demonstrate that DN T cell differentiation can proceed in the thymus and that DN T cell activation can occur independently of co-receptors.

Double negative T cells have not only been observed in quad-deficient mice. For instance, an increased number of DN T cells has been observed in many TCR transgenic models (Hillhouse and Lesage, 2012). In these TCR transgenic models, due to forced expression of an αβTCR transgene, the DN T cells do recognize peptide–MHC complexes in the absence of co-receptor expression, suggesting that they exhibit a high affinity for these antigenic complexes. Moreover, in the absence of CD28 co-stimulation which is required for clonal deletion of thymocytes (Punt et al., 1994; Kishimoto and Sprent, 1997; Lesage et al., 1997), some thymocytes that strongly recognize self-ligands survive negative selection and ultimately develop into mature thymic DN T cells (Pobezinsky et al., 2012). Together, these results suggest that DN T cells may exhibit a relatively strong affinity toward their cognate ligands. This property is reminiscent of CD4^+^ Foxp3^+^ regulatory T cells (Tregs) and NKT cells, two immunoregulatory T cell subsets which undergo agonist selection in the thymus (Baldwin et al., 2004).

In addition to their distinct phenotype, DN T cells also exhibit a unique antigen-specific immunoregulatory potential. Indeed, the immunoregulatory function of DN T cells was first identified almost 25 years ago, when Strober et al. (1989) successfully cloned DN T cells from mice and subsequently demonstrated that DN T cells mediate suppressor activity in a mixed-lymphocyte reaction (MLR). Subsequently, Dr. Zhang’s group was the first to reveal the antigen-specific immunoregulatory potential of DN T cells (Zhang et al., 2000). Specifically, they showed that DN T cells from 2C TCR transgenic mice suppress the proliferation and cytotoxic activity of 2C TCR CD8^+^ T cells *in vitro*, but not of CD8^+^ T cells carrying other antigen specificities (Zhang et al., 2000; Young and Zhang, 2002). Moreover, the antigen-specific property of 2C TCR DN T cells is conferred, at least in part, by their ability to acquire peptide–MHC complexes from antigen presenting cells (Zhang et al., 2000; Priatel et al., 2001; Young and Zhang, 2002; Ford McIntyre et al., 2008), a process known as trogocytosis (Joly and Hudrisier, 2003). Notably, the *in vitro* antigen-specific suppressive activity of 2C TCR DN T cells toward CD8^+^ T cells was replicated using non-transgenic mice and humans (Zhang et al., 2000; Young and Zhang, 2002; Fischer et al., 2005). Together, these observations described a unique antigen-specific mode of immunoregulation provided by DN T cells, leading to the antigen-specific elimination of CD8^+^ T cells (Zhang et al., 2000; Young and Zhang, 2002).

The immunoregulatory potential of DN T cells has since been shown to extend beyond T cells. Indeed, TCR transgenic and non-transgenic DN T cells can also inhibit NK cells (He et al., 2007; Su et al., 2012), B cells (Zhang et al., 2006; Hillhouse et al., 2010; Ford McIntyre et al., 2011), and dendritic cells (Gao et al., 2011). The combination of their distinct phenotypic characteristics and their unique antigen-specific immunoregulatory properties toward multiple cellular targets has prompted investigators to examine the role of DN T cells in various disease models. Herein, we will review the promising therapeutic potential of DN T cells in the context of various disease settings. More specifically, we will describe the impact of DN T cell transfer on the induction of graft tolerance and the prevention of autoimmunity as well as present their dual role in preventing graft-vs-host disease (GVHD) while promoting graft-vs-tumor (GvT) responses.

## GRAFT TOLERANCE

**FIGURE 1:**
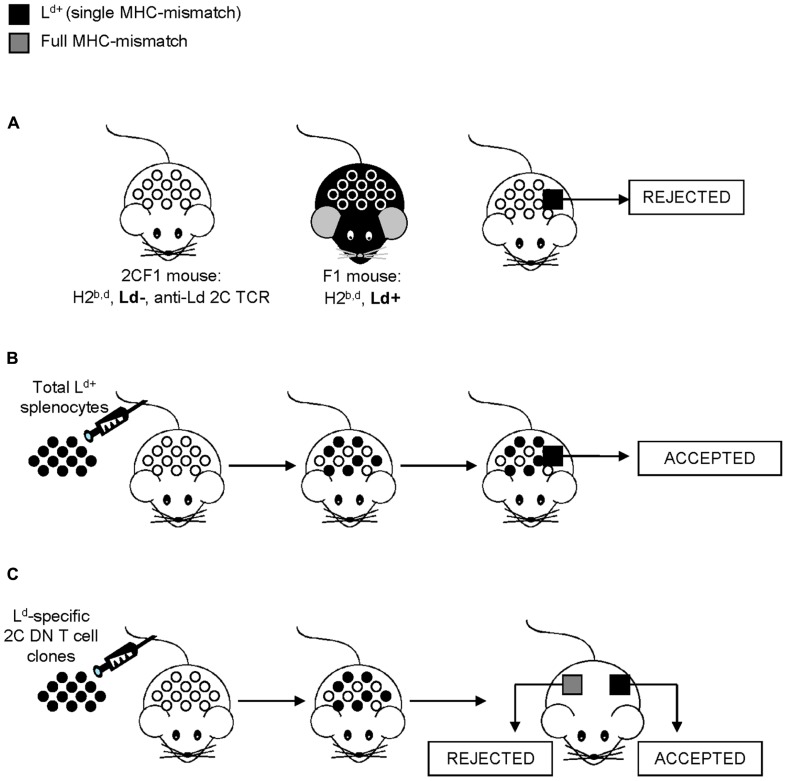
**Induction of allograft tolerance by the pre-transplantation infusion of donor-specific spleen cells or DN T cells**. This model takes advantage of the antigen-specific 2C TCR transgenic system and skin grafts to facilitate the study of allograft tolerance, where the 2C TCR transgene is alloreactive to the L^d^ MHC class I molecule. **(A)** C57BL/6 (H2^b^) mice are bred to BALB/c (H2^d^) mice to generate F1 mice (H2^b,d^) thus bearing the L^d^ MHC molecule (black mouse). C57BL/6 2C transgenic mice (H-2^b^) are bred to BALB/c (H2^*dm2*^) mice, which are a BALB/c L^d^ loss mutant (H2^d^, L^d^^-^; Rubocki et al., 1986), to generate 2CF1 mice (H2^b,d^, L^d^^-^) bearing the anti-L^d^ TCR (white mouse). Due to a single MHC-mismatch, skin grafts from L^d^^+^ F1 mice are rapidly rejected by the 2CF1 mice. **(B)** However, the transfer of donor spleen cells from L^d^^+^F1 mice to 2CF1 mice (L^d^^-^) prior to the L^d^^+^ skin graft transplantation results in graft survival. **(C)** Immunoregulatory 2C DN T cell clones obtained from 2CF1 TCR transgenic mice (L^d^^-^) prevent the rejection of single MHC-mismatch, allogeneic (L^d^^+^, in black) skin grafts. However, full MHC-mismatch, third party grafts (in gray) were rejected.

Although better known for their use in hematopoietic cell transplantation to establish donor chimerism or treat neoplastic relapse, donor leukocyte infusions (DLI) have also been shown to improve allograft survival after solid organ transplantation (Fehr and Sykes, 2004). Among possible mechanisms linking donor leukocyte transfer and allograft tolerance, DN T cells have been shown to increase allograft acceptance in various experimental settings. In an attempt to understand why DLI has a positive outcome on allograft survival, Dr. Zhang’s group took advantage of the antigen-specific 2C TCR transgenic model (Yang et al., 1998, 1999), where the 2C TCR is alloreactive to the L^d^ MHC class I molecule (Sha et al., 1988). Predictably, skin grafts bearing a single MHC-mismatch at L^d^ are thus rapidly rejected by the 2C TCR recipient mice due to the expression of L^d^ MHC class I molecule on the donor skin cells (Yang et al., 1998, 1999; **Figure 1A**). However, the injection of donor spleen cells to the 2C TCR recipient mice prior to the skin graft efficiently induced antigen-specific allograft tolerance (Yang et al., 1998, 1999; **Figure 1B**). The antigen-specific tolerance to skin allografts induced by the transfer of donor T cells was proposed to be mediated by 2C TCR transgenic DN T cells as only the 2C TCR transgenic DN T cell subset, but not the 2C CD4^+^ or 2C CD8^+^ T cell subset, was able to suppress an MLR response *in vitro* (Zhang et al., 2000). Accordingly, Zhang’s group showed that the injection of 2C TCR F1 DN T cell clones was sufficient to induce both prolonged survival of both skin and cardiac allografts (Zhang et al., 2000; Chen et al., 2003b; **Figure 1C**). Importantly, the allograft tolerance was antigen-specific, as full MHC-mismatched third party grafts were rapidly rejected (Zhang et al., 2000; **Figure 1C**). Collectively, these data demonstrate that 2C DN T cells are sufficient to induce both skin and cardiac allograft survival, suggesting that immunoregulatory DN T cells contribute to the benefits of DLI on allograft survival.

To further understand the mechanism by which 2C DN T cells promote antigen-specific allograft tolerance, Young et al. (2002) undertook the examination of the leukocytes found within the tolerated skin grafts. In doing so, they discovered that 2C DN T cells are the predominant leukocyte found within the accepted skin allografts. Moreover, the 2C DN T cells isolated from mice which had received donor spleen cells prior to the skin graft demonstrate an enhanced suppressive function toward 2C CD8^+^ T cells *in vitro* (Young et al., 2002). Finally, the comparison of transcriptome profiles between 2C TCR F1 DN T cells clones that are able or unable to confer cardiac allograft tolerance revealed FcRγ as a potential molecule involved in defining the tolerogenic potential of 2C DN T cells (Lee et al., 2005). The importance of FcRγ expression on 2C DN T cells for the induction of allograft tolerance was confirmed as the adoptive transfer of FcRγ-sufficient, but not FcRγ-deficient, 2C DN T cells prior to transplantation increased skin allograft survival (Thomson et al., 2006). Altogether, these results demonstrate that 2C DN T cells participate in allograft tolerance, likely by inhibiting pathogenic 2C CD8^+^ T cell responses, at least in the MHC class I-restricted graft tolerance model (Hillhouse and Lesage, 2012).

Importantly, these observations were not limited to the 2C TCR transgenic setting. Indeed, the injection of allogeneic donor spleen cells bearing a single MHC class I mismatch prior to skin transplantation in the non-transgenic setting also resulted in prolonged allograft survival (Yang et al., 1999; Young et al., 2002; Zhang et al., 2002). Analogous to the 2C TCR model, DN T cells from non-transgenic mice that are activated *in vivo* following the injection of donor spleen cells preferentially accumulate within the skin allograft and eliminate CD8^+^ T cells in an antigen-specific manner *in vitro* (Young et al., 2002). In a full MHC-mismatch heart allograft, injection of non-transgenic DN T cells further promoted rapamycin-induced graft tolerance (Zhang et al., 2011b). In addition, *in vitro*-generated DN T cells can also provide antigen-specific skin and pancreatic islet allograft tolerance (Zhang et al., 2007, 2011a). Moreover, with regards to xenografts, the injection of non-transgenic rat donor spleen cells in mice prevented CD4^+^ T cell-mediated cardiac xenograft rejection (Chen et al., 2003a, 2005). Similar to the transgenic setting, xenograft tolerance was induced by DN T cells, as the transfer of DN T cells that had been isolated from mice which had received xenogeneic donor spleen cells, was sufficient to ensure cardiac xenograft tolerance (Chen et al., 2003a). In this xenogeneic model, DN T cells efficiently suppressed the proliferation of both CD4^+^ and CD8^+^ T cell anti-donor reactive cells (Chen et al., 2003a, 2005). Using a variant of this cardiac xenograft model, it was also shown that DN T cells can eliminate B cells, leading to a reduction in anti-donor-specific antibody levels and delayed graft rejection (Ma et al., 2008). Taken together, these findings suggest that, as opposed to the MHC class I-restricted 2C TCR DN T cells, DN T cells from non-transgenic mice can suppress CD4^+^ and CD8^+^ T cells as well as B cells in an antigen-specific manner and potently suppress both skin and cardiac allograft, as well as cardiac xenograft, rejection.

Fas–FasL interactions have been proposed as the molecular mechanism by which DN T cells eliminate anti-donor T cells, while the elimination of B cells is considered to be perforin-mediated (Zhang et al., 2000, 2006; Priatel et al., 2001; Ma et al., 2008; Ford McIntyre et al., 2011). Interestingly, an accumulation of DN T cells is observed in both Fas- and FasL-deficient mice (Watanabe-Fukunaga et al., 1992; Takahashi et al., 1994), suggesting that the Fas–FasL pathway may regulate the number of DN T cells *in vivo*. By taking advantage of lpr mice, which exhibit a Fas-deficiency, Ford et al. (2002) demonstrated that DN T cells from lpr mice can mediate their immunoregulatory function through the Fas pathway as long as the target T cells express a functional Fas protein. Indeed, DN T cells from lpr mice could effectively delay both single MHC class I- and single MHC class II-mismatched skin allograft rejection (Ford et al., 2002). In summary, DN T cells can clearly induce an antigen-specific tolerance to both allografts and xenografts by eliminating or inhibiting the function of various target immune cells.

## AUTOIMMUNITY

The antigen-specific immunoregulatory potential of DN T cells in graft tolerance suggests that they may also participate in the induction of immune tolerance in various autoimmune settings. As a result, the role of DN T cells has been mostly explored in autoimmune lymphoproliferative syndrome (ALPS), systemic lupus erythematosus (SLE) and mouse models of type 1 diabetes (T1D), as described below.

### AUTOIMMUNE LYMPHOPROLIFERATIVE SYNDROME

Autoimmune lymphoproliferative syndrome is a rare disorder characterized by mutations in either Fas or FasL (Rieux-Laucat et al., 1995; Del-Rey et al., 2006), which results in defective Fas-mediated apoptosis and, consequently, the abnormal accumulation of activated lymphocytes. ALPS patients on average present with a 10-fold increase in DN T cell proportion (Bleesing et al., 2001a; Bristeau-Leprince et al., 2008; Magerus-Chatinet et al., 2009) and a sizeable proportion of DN T cells can also be found in the lymphoid organs of the murine models for ALPS, namely *lpr/lpr* (Fas-deficient) and *gld/gld* (FasL-deficient) mice (Cohen and Eisenberg, 1992; Watanabe-Fukunaga et al., 1992; Takahashi et al., 1994). Moreover, a greater proportion of DN T cells from ALPS patients, in comparison to healthy controls, express the B cell antigen, B220 (Bleesing et al., 2001a), which is consistent with findings in *lpr/lpr* mice (Davidson et al., 1986). Accordingly, B220 expression is a general feature of proliferating T cells (Bleesing et al., 2001b), which explains why B220^+^ DN T cells can also be found in healthy individuals. Although it cannot be denied that a drastic increase in the proportion of DN T cells can be observed in *lpr/lpr* and *gld/gld* mice, as well as ALPS patients, there is arguably no evidence to suggest that the accumulation of DN T cells is pathogenic. Indeed, Fas-deficient DN T cells found in *lpr/lpr* mice, but not FasL-deficient DN T cells found in *gld/gld* mice, remain functional and, as mentioned above, can efficiently induce graft tolerance (Ford et al., 2002). Hence, as DN T cell-mediated suppression depends on interactions between FasL on DN T cells and Fas on target T cells (Ford et al., 2002), it has been suggested that DN T cells accumulate in an attempt to compensate for their inability to suppress autoimmune T cells in *lpr/lpr* mice (Ford et al., 2002).

### SYSTEMIC LUPUS ERYTHEMATOSUS

Similar to ALPS patients, DN T cells are also found in greater numbers in the peripheral blood of patients with SLE (Crispin et al., 2008). However, in the case of SLE, these DN T cells are major producers of IL-17 (Crispin and Tsokos, 2009), which is pathogenic in this disease (Wong et al., 2000; Doreau et al., 2009). Moreover, IL-17-producing DN T cells can be found in the kidney sections of patients with lupus nephritis (Crispin et al., 2008). These results lead Crispin et al. (2008) to suggest that DN T cells themselves are pathogenic in SLE. Yet, it must be noted that, in these studies, DN T cells were defined as CD4^-^CD8^-^TCRαβ^+^ cells, which may contain contaminating CD4^-^CD8^-^ NKT cells expressing a TCR restricted to CD1d molecules (Hillhouse and Lesage, 2012). Of relevance, NKT cells do indeed include a subset of CD4^-^NK1.1^-^ IL-17-producing cells (Coquet et al., 2008). Moreover, NKT cells have been implicated in SLE pathogenesis where the activation of NKT cells through the administration of α-GalCer exacerbated disease whereas the inhibition of NKT cell activation using an anti-CD1d blocking antibody resulted in disease amelioration (Zeng et al., 2003). Thus, NKT cells cannot be ruled out as the true IL-17-producing cell subset in this particular study. Nevertheless, IL-17-producing DN T cells have also been shown to play a protective role against bacterial infections (Cowley et al., 2005; Riol-Blanco et al., 2010). Altogether, an uncertain, yet possible, role for DN T cells in patients with SLE remains to be defined.

### TYPE 1 DIABETES

Type 1 diabetes occurs as a result of the antigen-specific elimination of pancreatic insulin-producing β cells. It thus presents as a relevant model to investigate the antigen-specific immunoregulatory potential of DN T cells.

The tolerogenic role of DN T cells in the prevention of T1D was first revealed using the P14/RIP-gp transgenic mouse model (Ohashi et al., 1991), where the MHC class I-restricted P14 TCR transgene recognizes the lymphocytic choriomeningitis virus (LCMV) protein, gp33–41 (gp33), which is expressed under the rat insulin promoter (RIP) forcing its expression in the pancreatic tissue. RIP-gp mice that are crossed to P14 mice carry a very high percentage of gp33-reactive T cells, yet the resulting P14 TCR:RIP-gp mice do not spontaneously develop diabetes on the C57BL/6 background (Ohashi et al., 1991). Indeed, P14/RIP-gp transgenic mice rapidly develop diabetes upon treatment with gp33 peptide and anti-CD40 agonistic antibody, which results in the infiltration of pancreatic islets by activated CD8^+^ T cells and the subsequent destruction of the insulin-producing β cells (Garza et al., 2000). Using this model, it was shown that the transfer of gp33 activated P14 DN T cells 1 day prior to diabetes induction can inhibit diabetes development (Ford et al., 2007). These results were the first indication that DN T cells may participate in the prevention of a CD8^+^ T cell-driven T1D pathology, further lending support for a potential role for DN T cells toward the elimination of CD8^+^ T cells *in vivo*.

The protective role of DN T cells in diabetes development was further investigated by our group using the 3A9 TCR:insHEL transgenic system, in which the MHC class II-restricted 3A9 TCR transgene recognizes a peptide from hen egg lysozyme (HEL) presented by I-A^k^, while the insHEL transgene forces the expression of HEL in the pancreatic tissue. Although TCR:insHEL BALB.K mice are relatively resistant to T1D, CD47-deficient TCR:insHEL BALB.K mice have a high and spontaneous incidence of diabetes. Using this model of spontaneous T1D, a single transfer of 3A9 DN T cells in the TCR:insHEL CD47-deficient BALB.K transgenic model was able to significantly inhibit the development of T1D (Dugas et al., 2010). Notably, a 3A9 DN T cell transfer led to a significant reduction in antigen-specific autoantibody serum levels (Dugas et al., 2010), suggesting the *in vivo* elimination of autoreactive B cells. We subsequently demonstrated that 3A9 DN T cells efficiently eliminate HEL-loaded B cells *in vitro* (Hillhouse et al., 2010). Although this model is imperfect in that it is a CD47-deficient model, where CD47 is implicated in apoptosis, phagocytosis, cell migration, and T cell responses (Oldenborg, 2004; Chao et al., 2012), it is nevertheless a spontaneous model of T1D wherein the results complement the findings of Ford et al. (2007) suggesting that DN T cells may be of therapeutic interest for T1D.

Interestingly, the proportion of DN T cells is significantly reduced in diabetes-prone mice in comparison to diabetes-resistant mice in both the transgenic and non-transgenic systems (Dugas et al., 2010). It is of relevance that both the non-transgenic and TCR:insHEL transgenic diabetes-prone non-obese diabetic (NOD) mouse models exhibit a low number of DN T cells relative to other diabetes-resistant strains as it suggests that low DN T cell numbers are associated with diabetes susceptibility. Despite a reduction in cell number, 3A9 DN T cells from 3A9 TCR transgenic NOD. H2^*k*^ mice carrying a diabetes-prone genetic background (Lesage et al., 2002) exhibit an equally potent cytotoxic function in comparison to 3A9 DN T cells from diabetes-resistant 3A9 TCR transgenic B10.Br mice (Hillhouse et al., 2010). Therefore, the association of DN T cells with diabetes susceptibility is due to a deficiency in DN T cell number rather than function. Altogether, these results further suggest that the restoration of DN T cell number can prevent T1D development in otherwise diabetes-susceptible mice.

The role of DN T cells in the prevention of T1D was also recently evaluated using non-transgenic DN T cells. Indeed, Duncan et al. (2010) have demonstrated that non-transgenic DN T cells can protect from autoimmune diabetes. Specifically, diabetes induction was inhibited if DN T cells were transferred into recipient NOD.SCID (severe combined immunodeficiency) mice 1 month prior to co-infusion with diabetogenic spleen cells, whereas the simultaneous co-infusion of diabetogenic spleen cells and DN T cells does not protect from disease (Duncan et al., 2010). However, as NKT cells were not fully excluded from the cellular preparation, additional studies using NKT cell-depleted non-transgenic DN T cells are warranted to firmly establish the biological function of these cells in the prevention of autoimmune diabetes in diabetes-susceptible NOD mice. Notably, further investigation is still needed to verify the role of DN T cells in T1D development using non-lymphopenic NOD mice, thus under more physiological and spontaneous conditions. Nevertheless, these results do provide useful information regarding the critical time of treatment initiation as well as evidence that non-transgenic DN T cells may exhibit the potential to inhibit T1D development in non-transgenic mice.

A more recent study further evaluated the role of non-transgenic DN T cells in T1D. Here, it was shown that 50% of CD4^+^ T cells isolated from an MLR had been converted to CD4^-^CD8^-^ T cells. These CD4^+^ T cell-converted DN T cells sorted by flow cytometry were shown to delay T1D onset when adoptively transferred to NOD.SCID mice in combination with diabetogenic T cells (Zhang et al., 2011a). This delay was further enhanced when using GAD65 antigen to stimulate the CD4^+^ T cells, thereby likely generating a higher proportion of GAD65-specific DN T cells. Moreover, a single transfer of GAD65-specific DN T cells specificity was able to prevent diabetes development in 5-week-old NOD mice and decrease blood glucose levels in new-onset diabetic NOD mice (Zhang et al., 2011a). Therefore, this study offers a potentially translatable therapeutic approach for the generation of antigen-specific DNT cells in the prevention and treatment of T1D.

Altogether, these findings point toward an antigen-specific immunoregulatory role for DN T cells in autoimmune diseases. The therapeutic potential of these cells certainly merits further investigation in additional pre-clinical models.

## GRAFT-vs-HOST DISEASE AND CANCER

In the treatment of hematological malignancies, allogeneic hematopoietic cell transplantation (AHCT) can eradicate several blood cancers that are incurable by chemotherapy alone. Despite indisputable successes, the efficacy of AHCT is still limited by cancer recurrence and the development of GVHD (Raiola et al., 2003). While the acute form of GVHD is triggered by direct T cell recognition of histocompatibility antigens, the pathophysiology of chronic GVHD remains more elusive and relies on several immune cell types (Shlomchik, 2007). Currently, 40–80% of patients develop chronic GVHD after AHCT, which brings substantial morbidity and mortality (Lee, 2005). Several lines of evidence suggest that immunoregulatory immune cells are paramount to GVHD prevention. Indeed, both CD4^+^ Tregs and NKT cells have been suggested as major contributors of allotolerance in hematopoietic cell transplantation (Hoffmann et al., 2002; Kohrt et al., 2010; Di Ianni et al., 2011; Chaidos et al., 2012). However, the ability of Tregs to maintain their anti-tumor T cell responses remains controversial (Onizuka et al., 1999; Shimizu et al., 1999; Liyanage et al., 2002; Somasundaram et al., 2002; Kohrt et al., 2010). Here, we review the potential of DN T cells at inhibiting GVHD while specifically promoting anti-tumor responses.

### GRAFT-vs-HOST DISEASE

**FIGURE 2:**
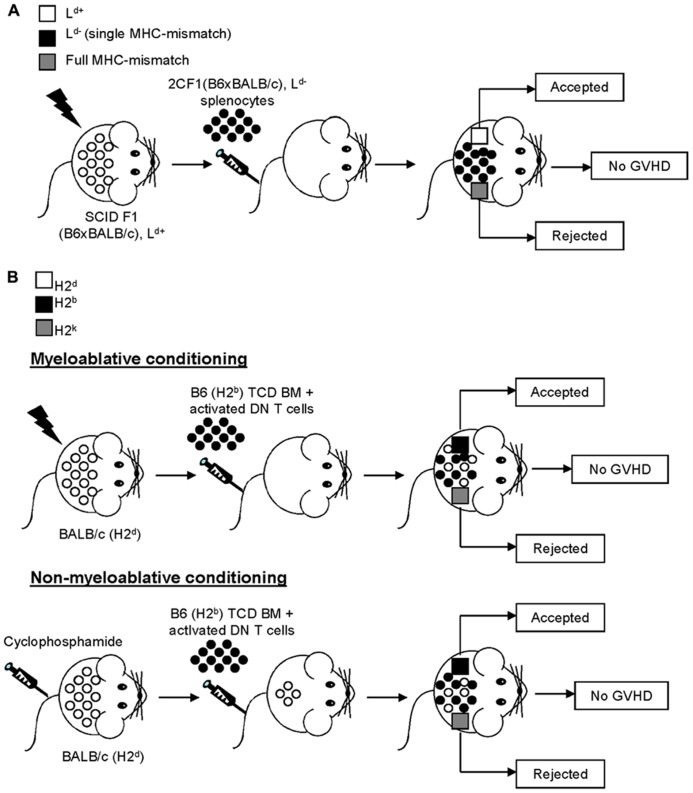
**Double negative T cells promote tolerance while inhibiting GVHD**. **(A)** Immunodeficient SCID F1 mice (H2^b,d^, L^d^^+^) were sublethally irradiated and reconstituted with 2CF1 (H2^b,dm2^, L^d^^-^) L^d^-specific spleen cells. Three weeks later, recipient mice were given skin grafts from F1 (L^d^^+^, in white) and full MHC-mismatch, third party (in gray) mice. The third party skin graft was rejected, whereas the skin graft from the F1 (L^d^^+^) donor mouse was accepted. Importantly, the recipient mice showed no signs of GVHD and exhibited a prominent increase in the levels of donor-derived DN T cells over time. **(B)** BALB/c (H2^d^) mice underwent myeloablative (*top panel*, sublethal irradiation) or non-myeloablative (*bottom panel*, busulfan treatment) conditioning prior to being reconstituted with a combination of C57BL/6 (H2^b^) DN T cells and T cell-depleted (TCD) C57BL/6 (H2^b^) bone marrow (BM) cells. Subsequently, recipient mice received skin grafts from donor C57BL/6 (H2^b^, in black) and third party (H2^k^, in gray) mice. Regardless of the type of conditioning regimen utilized, the recipient mice accepted all C57BL/6 (H2^b^, in black) donor grafts while third party (H2^k^, in gray) grafts were rejected.

Mouse models of GVHD, although they admittedly do not fully replicate human disease, facilitate the examination of the cellular process amid the allogeneic responses which give rise to a GVHD-like pathology *in vivo* (Shlomchik, 2007). A potential role for DN T cells in the prevention of GVHD can be traced back to over two decades ago when Bruley-Rosset et al. (1990) demonstrated that transfer of spleen cells from pre-immunized mice was able to inhibit GVHD only when DN T cells were included in the cellular preparations. Although the DN T cells were not void of contaminating NKT cells, these results still suggested that DN T cells play a role in GVHD prevention. Because of their prominent antigen-specific immunoregulatory potential as well as their ability to promote allotolerance, the contribution of DN T cells at preventing GVHD was later investigated using the 2C TCR single MHC-mismatch model (Young et al., 2003a), for which DN T cells have been shown to effectively prevent allograft rejection (Zhang et al., 2000; Young et al., 2002; Chen et al., 2003b; **Figure 1**). Specifically, mice bearing a single MHC-mismatch (L^d^) injected with L^d^-specific 2C TCR transgenic spleen cells survived for more than 150 days without any clinical or histological signs of acute or chronic GVHD (Young et al., 2003a; **Figure 2A**). The tolerance of donor spleen cells may be explained, at least in part, by the accumulation of 2C TCR DN T cells, which can effectively inhibit pathogenic CD8^+^ T cell responses, in recipient mice (Young et al., 2003a). Subsequently, the role of non-transgenic DN T cells in both parent to F1 and fully MHC-mismatched bone marrow (BM) transplantation following either a myeloablative (He et al., 2007) or non-myeloablative regimen (Su et al., 2012) was investigated (**Figure 2B**). In stark contrast to CD4^+^ and CD8^+^ T cells, the co-injection of non-transgenic DN T cells with T cell-depleted BM into a sublethally irradiated host ensured prolonged recipient survival in the absence of clinical signs of GVHD (He et al., 2007; Su et al., 2012; **Figure 2B**), indicating that DN T cells are not alloreactive nor pathogenic in this context. Moreover, this led to stable mixed chimerism and, as opposed to BM transplantation alone, promoted donor allotolerance (He et al., 2007; Su et al., 2012; **Figure 2B**). Together, these findings demonstrate that, as opposed to CD4^+^ or CD8^+^ T cells, the transfer of allogeneic DN T cells does not cause GVHD. Rather, DN T cells prevent GVHD, induce mixed chimerism and promote donor-specific allotolerance.

Notably, there is accrued evidence that DN T cells also prevent GVHD in humans upon AHCT. Indeed, the stem cells used for AHCT are currently most commonly obtained from the peripheral blood mononuclear cells of granulocyte colony-stimulating factor (G-CSF)-treated donors, where G-CSF is used to mobilize CD34^+^ hematopoietic stem cells into the blood (Shlomchik, 2007). Interestingly, in addition to skewing toward a Th2 phenotype, G-CSF treatment also results in an increase in the proportion of immunoregulatory DN T cells in the blood (Kusnierz-Glaz et al., 1997). The combination of these two phenotypes may explain why the transfer of CD34^+^ stem cells isolated following G-CSF treatment does not increase the incidence of acute GVHD relative to classical BM transplants. More recently, two studies also noted a clear correlation between an elevated proportion of DN T cells and a low incidence of GVHD development following AHCT (McIver et al., 2008; Ye et al., 2011). Together, these observations support the view that human DN T cells may prevent GVHD.

### THE ANTI-TUMOR RESPONSE

In addition to preventing GVHD, DN T cells demonstrate an anti-tumor activity without causing GVHD in recipient mice (Young et al., 2001, 2003b). Indeed, >90% of sublethally irradiated immunodeficient SCID mice that were co-infused with a lethal dose of A20 lymphoma cells together with allogeneic 2C TCR transgenic spleen cells survived indefinitely in the absence of GVHD. Interestingly, recipient mice exhibited a 15-fold increase in the number of DN T cells in their spleen (Young et al., 2003b), suggesting an association between DN T cells and the inhibition of GVHD as well as tumor growth. Accordingly, Young et al. (2003b) demonstrated that the injection of either 2C DN T cell clones or non-transgenic DN T cells was sufficient to prevent A20 lymphoma tumor growth, without inducing clinical or histological signs of GVHD. Altogether, these results further demonstrate the anti-tumoral potential of DN T cells.

To further define the potential use of DN T cells in the treatment of hematological malignancies, Merims et al. (2011) performed a pre-clinical study in acute myeloid leukemia (AML) patients undergoing complete remission following standard chemotherapy. In this study, they demonstrate that DN T cells can be readily isolated from the blood of AML patients. Moreover, the isolated DN T cells maintain their phenotype following a proficient cellular expansion reaching nearly 500-fold in 2 weeks (Merims et al., 2011). Of interest, the DN T cells isolated and expanded *in vitro* demonstrated efficient cytolytic activity toward the autologous leukemic cells in six out of seven patients (Merims et al., 2011). Importantly, the anti-tumoral activity of DN T cells may extend beyond hematological malignancies, as melanoma-reactive DN T cells have also been successfully isolated from a previously immunized melanoma patient (Voelkl et al., 2009). The mechanism by which DN T cells are able to confer antigen-specific immune tolerance all the while providing an anti-tumor response is counterintuitive and certainly merits further investigation.

## CONCLUSION

Immunoregulatory DN T cells, which compose approximately 1–3% of T cells in human PBMCs (Fischer et al., 2005), demonstrate a unique antigen-specific therapeutic potential, such that they provide a favorable outcome in graft tolerance, autoimmunity and GVHD. In addition, DN T cells exhibit an interesting anti-tumoral potential. This seemingly broad applicability of DN T cells at conferring antigen-specific immune tolerance must be interpreted with caution. Indeed, DN T cells compose a heterogeneous subset of cells, which include all CD4^-^CD8^-^CD3^+^ T cell subsets, namely TCRγδ T cells, NKT cells and immunoregulatory TCRαβ DN T cells (Hillhouse and Lesage, 2012). Despite the heterogeneity in the description of DN T cells in various studies, which do not always exclude TCRγδ and/or NKT cells, most studies in TCR transgenic and non-transgenic mice as well as in humans point toward an immunoregulatory role for DN T cells. A few notable exceptions to this are the description of pathogenic IL-17-producing DN T cells in SLE (Crispin and Tsokos, 2009) and the characterization of IL-23 responsive DN T cells controlling bacterial infections (Cowley et al., 2005, 2010; Riol-Blanco et al., 2010). Clearly, additional studies aimed at identifying unique markers for these unique cell types will help clarify which specific cellular subset contributes to each specific biological setting.

In conclusion, immunoregulatory DN T cells exhibit great potential as a cellular therapy for various models of disease, with exciting advances in the pre-clinical setting. As the function of DN T cells is mainly antigen-specific, the use of DN T cells in immunotherapy should lead to fewer side-effects and a decreased risk of infections, which is a major concern in the application of broad immunosuppressive regimens. Moreover, DN T cells exhibit a potent immunosuppressive potential in multiple model systems, from transplantation to autoimmunity, suggesting a vast array of applicability of DN T cells in cellular therapy.

## Conflict of Interest Statement

The authors declare that the research was conducted in the absence of any commercial or financial relationships that could be construed as a potential conflict of interest.

## References

[B1] Abraham V. S. Sachs D. H. Sykes M. (1992). Mechanism of protection from graft-versus-host disease mortality by IL-2. III. Early reductions in donor T cell subsets and expansion of a CD3^+^CD4^-^CD8^-^ cell population. *J. Immunol.* 148 3746–3752 1534824

[B2] Baldwin T. A. Hogquist K. A. Jameson S. C. (2004). The fourth way? Harnessing aggressive tendencies in the thymus. *J. Immunol.* 173 6515–6520 15557139 10.4049/jimmunol.173.11.6515

[B3] Bleesing J. J. Brown M. R. Dale J. K. Straus S. E. Lenardo M. J. Puck J. M. (2001a). TcR-alpha/beta(+) CD4(-)CD8(-) T cells in humans with the autoimmune lymphoproliferative syndrome express a novel CD45 isoform that is analogous to murine B220 and represents a marker of altered *O*-glycan biosynthesis. *Clin. Immunol.* 100 314–324 11513545 10.1006/clim.2001.5069

[B4] Bleesing J. J. Morrow M. R. Uzel G. Fleisher T. A. (2001b). Human T cell activation induces the expression of a novel CD45 isoform that is analogous to murine B220 and is associated with altered *O*-glycan synthesis and onset of apoptosis. *Cell. Immunol.* 213 72–81 11747358 10.1006/cimm.2001.1865

[B5] Bristeau-Leprince A. Mateo V. Lim A. Magerus-Chatinet A. Solary E. Fischer A. (2008). Human TCR alpha/beta^+^ CD4^-^CD8^-^ double-negative T cells in patients with autoimmune lymphoproliferative syndrome express restricted Vbeta TCR diversity and are clonally related to CD8^+^ T cells. *J. Immunol.* 181 440–448 18566410 10.4049/jimmunol.181.1.440

[B6] Bruley-Rosset M. Miconnet I. Canon C. Halle-Pannenko O. (1990). Mlsa generated suppressor cells. I. Suppression is mediated by double-negative (CD3^+^CD5^+^CD4^-^CD8^-^) alpha/beta T cell receptor-bearing cells. *J. Immunol.* 145 4046–4052 1701781

[B7] Chaidos A. Patterson S. Szydlo R. Chaudhry M. S. Dazzi F. Kanfer E. (2012). Graft invariant natural killer T-cell dose predicts risk of acute graft-versus-host disease in allogeneic hematopoietic stem cell transplantation. *Blood* 119 5030–5036 22371885 10.1182/blood-2011-11-389304 PMC6143158

[B8] Chao M. P. Weissman I. L. Majeti R. (2012). The CD47-SIRPalpha pathway in cancer immune evasion and potential therapeutic implications. *Curr. Opin. Immunol.* 24 225–232 22310103 10.1016/j.coi.2012.01.010 PMC3319521

[B9] Chen W. Ford M. S. Young K. J. Cybulsky M. I. Zhang L. (2003a). Role of double-negative regulatory T cells in long-term cardiac xenograft survival. *J. Immunol.* 170 1846–1853 12574350 10.4049/jimmunol.170.4.1846

[B10] Chen W. Ford M. S. Young K. J. Zhang L. (2003b). Infusion of in vitro-generated DN T regulatory cells induces permanent cardiac allograft survival in mice. *Transplant. Proc.* 35 2479–2480 14611991 10.1016/j.transproceed.2003.08.030

[B11] Chen W. Zhou D. Torrealba J. R. Waddell T. K. Grant D. Zhang L. (2005). Donor lymphocyte infusion induces long-term donor-specific cardiac xenograft survival through activation of recipient double-negative regulatory T cells. *J. Immunol.* 175 3409–3416 16116235 10.4049/jimmunol.175.5.3409

[B12] Cohen P. L. Eisenberg R. A. (1992). The lpr and gld genes in systemic autoimmunity: life and death in the Fas lane. *Immunol. Today* 13 427–428 1282318 10.1016/0167-5699(92)90066-G

[B13] Coquet J. M. Chakravarti S. Kyparissoudis K. Mcnab F. W. Pitt L. A. Mckenzie B. S. (2008). Diverse cytokine production by NKT cell subsets and identification of an IL-17-producing CD4^-^NK1.1^-^ NKT cell population. * Proc. Natl. Acad. Sci. U.S.A.* 105 11287–11292 18685112 10.1073/pnas.0801631105 PMC2516267

[B14] Cowley S. C. Hamilton E. Frelinger J. A. Su J. Forman J. Elkins K. L. (2005). CD4^-^CD8^-^ T cells control intracellular bacterial infections both in vitro and in vivo. *J. Exp. Med.* 202 309–319 16027239 10.1084/jem.20050569 PMC2212999

[B15] Cowley S. C. Meierovics A. I. Frelinger J. A. Iwakura Y. Elkins K. L. (2010). Lung CD4^-^CD8^-^ double-negative T cells are prominent producers of IL-17A and IFN-gamma during primary respiratory murine infection with *Francisella tularensis* live vaccine strain. *J. Immunol.* 184 5791–5801 20393138 10.4049/jimmunol.1000362

[B16] Crispin J. C. Oukka M. Bayliss G. Cohen R. A. Van Beek C. A. Stillman I. E. (2008). Expanded double negative T cells in patients with systemic lupus erythematosus produce IL-17 and infiltrate the kidneys. *J. Immunol.* 181 8761–8766 19050297 10.4049/jimmunol.181.12.8761 PMC2596652

[B17] Crispin J. C. Tsokos G. C. (2009). Human TCR-alpha beta^+^ CD4^-^ CD8^-^ T cells can derive from CD8^+^ T cells and display an inflammatory effector phenotype. *J. Immunol.* 183 4675–4681 19734235 10.4049/jimmunol.0901533 PMC2878279

[B18] Davidson W. F. Dumont F. J. Bedigian H. G. Fowlkes B. J Morse H. C. III. (1986). Phenotypic, functional, and molecular genetic comparisons of the abnormal lymphoid cells of C3H-lpr/lpr and C3H-gld/gld mice. *J. Immunol.* 136 4075–4084 3009614

[B19] Del-Rey M. Ruiz-Contreras J. Bosque A. Calleja S. Gomez-Rial J. Roldan E. (2006). A homozygous Fas ligand gene mutation in a patient causes a new type of autoimmune lymphoproliferative syndrome. *Blood* 108 1306–1312 16627752 10.1182/blood-2006-04-015776

[B20] Di Ianni M. Falzetti F. Carotti A. Terenzi A. Castellino F. Bonifacio E. (2011). Tregs prevent GVHD and promote immune reconstitution in HLA-haploidentical transplantation. *Blood* 117 3921–3928 21292771 10.1182/blood-2010-10-311894

[B21] Doreau A. Belot A. Bastid J. Riche B. Trescol-Biemont M. C. Ranchin B. (2009). Interleukin 17 acts in synergy with B cell-activating factor to influence B cell biology and the pathophysiology of systemic lupus erythematosus. *Nat. Immunol.* 10 778–785 19483719 10.1038/ni.1741

[B22] Dugas V. Beauchamp C. Chabot-Roy G. Hillhouse E. E. Lesage S. (2010). Implication of the CD47 pathway in autoimmune diabetes. *J. Autoimmun.* 35 23–32 20138735 10.1016/j.jaut.2010.01.002

[B23] Duncan B. Nazarov-Stoica C. Surls J. Kehl M. Bona C. Casares S. (2010). Double negative (CD3^+^4^-^8^-^) TCRαβ splenic cells from young NOD mice provide long-lasting protection against type 1 diabetes. *PLoS ONE * 5:e11427 10.1371/journal.pone.0011427 PMC2896421 20625402

[B24] Fehr T. Sykes M. (2004). Tolerance induction in clinical transplantation. *Transpl. Immunol.* 13 117–130 15380542 10.1016/j.trim.2004.05.009

[B25] Fischer K. Voelkl S. Heymann J. Przybylski G. K. Mondal K. Laumer M. (2005). Isolation and characterization of human antigen-specific TCR alpha beta^+^ CD4(-)CD8- double-negative regulatory T cells. *Blood* 105 2828–2835 15572590 10.1182/blood-2004-07-2583

[B26] Ford M. S. Chen W. Wong S. Li C. Vanama R. Elford A. R. (2007). Peptide-activated double-negative T cells can prevent autoimmune type-1 diabetes development. *Eur. J. Immunol.* 37 2234–2241 17578845 10.1002/eji.200636991

[B27] Ford M. S. Young K. J. Zhang Z. Ohashi P. S. Zhang L. (2002). The immune regulatory function of lymphoproliferative double negative T cells in vitro and in vivo. *J. Exp. Med.* 196 261–267 12119351 10.1084/jem.20020029 PMC2193928

[B28] Ford McIntyre M. S. Gao J. F. Li X. Naeini B. M. Zhang L. (2011). Consequences of double negative regulatory T cell and antigen presenting cell interaction on immune response suppression. *Int. Immunopharmacol.* 11 597–603 21109036 10.1016/j.intimp.2010.11.015

[B29] Ford McIntyre M. S. Young K. J. Gao J. Joe B. Zhang L. (2008). Cutting edge: in vivo trogocytosis as a mechanism of double negative regulatory T cell-mediated antigen-specific suppression. *J. Immunol.* 181 2271–2275 18684915 10.4049/jimmunol.181.4.2271

[B30] Gao J. F. McIntyre M. S. Juvet S. C. Diao J. Li X. Vanama R. B. (2011). Regulation of antigen-expressing dendritic cells by double negative regulatory T cells. *Eur. J. Immunol.* 41 2699–2708 21660936 10.1002/eji.201141428

[B31] Garza K. M. Chan S. M. Suri R. Nguyen L. T. Odermatt B. Schoenberger S. P. (2000). Role of antigen-presenting cells in mediating tolerance and autoimmunity. *J. Exp. Med.* 191 2021–2027 10839816 10.1084/jem.191.11.2021 PMC2213533

[B32] He K. M. Ma Y. Wang S. Min W. P. Zhong R. Jevnikar A. (2007). Donor double-negative Treg promote allogeneic mixed chimerism and tolerance. *Eur. J. Immunol.* 37 3455–3466 18000953 10.1002/eji.200737408

[B33] Hillhouse E. E. Beauchamp C. Chabot-Roy G. Dugas V. Lesage S. (2010). Interleukin-10 limits the expansion of immunoregulatory CD4^-^CD8^-^ T cells in autoimmune-prone non-obese diabetic mice. *Immunol. Cell Biol.* 88 771–780 20603635 10.1038/icb.2010.84

[B34] Hillhouse E. E. Lesage S. (2012). A comprehensive review of the phenotype and function of antigen-specific immunoregulatory double negative T cells. *J. Autoimmun.* 10.1016/j.jaut.2012.1007.1010 [Epub ahead of print] 22910322

[B35] Hoffmann P. Ermann J. Edinger M. Fathman C. G. Strober S. (2002). Donor-type CD4(+)CD25(+) regulatory T cells suppress lethal acute graft-versus-host disease after allogeneic bone marrow transplantation. *J. Exp. Med.* 196 389–399 12163567 10.1084/jem.20020399 PMC2193938

[B36] Joly E. Hudrisier D. (2003). What is trogocytosis and what is its purpose? *Nat. Immunol.* 4 815 10.1038/ni0903-815 12942076

[B37] Kishimoto H. Sprent J. (1997). Negative selection in the thymus includes semimature T cells. *J. Exp. Med.* 185 263–271 9016875 10.1084/jem.185.2.263 PMC2196120

[B38] Kohrt H. E. Pillai A. B. Lowsky R. Strober S. (2010). NKT cells, Treg, and their interactions in bone marrow transplantation. *Eur. J. Immunol.* 40 1862–1869 20583031 10.1002/eji.201040394 PMC2926162

[B39] Kusnierz-Glaz C. R. Still B. J. Amano M. Zukor J. D. Negrin R. S. Blume K. G. (1997). Granulocyte colony-stimulating factor-induced comobilization of CD4- CD8- T cells and hematopoietic progenitor cells (CD34+) in the blood of normal donors. *Blood* 89 2586–2595 9116306

[B40] Lee B. P. Mansfield E. Hsieh S. C. Hernandez-Boussard T. Chen W. Thomson C. W. (2005). Expression profiling of murine double-negative regulatory T cells suggest mechanisms for prolonged cardiac allograft survival. *J. Immunol.* 174 4535–4544 15814674 10.4049/jimmunol.174.8.4535

[B41] Lee S. J. (2005). New approaches for preventing and treating chronic graft-versus-host disease. *Blood* 105 4200–4206 15701727 10.1182/blood-2004-10-4023 PMC1895039

[B42] Lesage S. Charron J. Winslow G. Hugo P. (1997). Induction of thymocyte deletion by purified single peptide/major histocompatibility complex ligands. *J. Immunol.* 159 2078–2081 9278291

[B43] Lesage S. Hartley S. B. Akkaraju S. Wilson J. Townsend M. Goodnow C. C. (2002). Failure to censor forbidden clones of CD4 T cells in autoimmune diabetes. *J. Exp. Med.* 196 1175–1188 12417628 10.1084/jem.20020735 PMC2194101

[B44] Liyanage U. K. Moore T. T. Joo H. G. Tanaka Y. Herrmann V. Doherty G. (2002). Prevalence of regulatory T cells is increased in peripheral blood and tumor microenvironment of patients with pancreas or breast adenocarcinoma. *J. Immunol.* 169 2756–2761 12193750 10.4049/jimmunol.169.5.2756

[B45] Ma Y. He K. M. Garcia B. Min W. Jevnikar A. Zhang Z. X. (2008). Adoptive transfer of double negative T regulatory cells induces B-cell death in vivo and alters rejection pattern of rat-to-mouse heart transplantation. *Xenotransplantation* 15 56–63 18333914 10.1111/j.1399-3089.2008.00444.x

[B46] Magerus-Chatinet A. Stolzenberg M. C. Loffredo M. S. Neven B. Schaffner C. Ducrot N. (2009). FAS-L, IL-10, and double-negative CD4- CD8- TCR alpha/beta+ T cells are reliable markers of autoimmune lymphoproliferative syndrome (ALPS) associated with FAS loss of function. *Blood* 113 3027–3030 19176318 10.1182/blood-2008-09-179630

[B47] McIver Z. Serio B. Dunbar A. O’Keefe C. L. Powers J. Wlodarski M. (2008). Double-negative regulatory T cells induce allotolerance when expanded after allogeneic haematopoietic stem cell transplantation. *Br. J. Haematol.* 141 170–178 18318770 10.1111/j.1365-2141.2008.07021.x

[B48] Merims S. Li X. Joe B. Dokouhaki P. Han M. Childs R. W. (2011). Anti-leukemia effect of ex vivo expanded DNT cells from AML patients: a potential novel autologous T-cell adoptive immunotherapy. *Leukemia* 25 1415–1422 21566657 10.1038/leu.2011.99 PMC4214360

[B49] Ohashi P. S. Oehen S. Buerki K. Pircher H. Ohashi C. T. Odermatt B. (1991). Ablation of “tolerance” and induction of diabetes by virus infection in viral antigen transgenic mice. *Cell* 65 305–317 1901764 10.1016/0092-8674(91)90164-t

[B50] Oldenborg P. A. (2004). Role of CD47 in erythroid cells and in autoimmunity. *Leuk. Lymphoma* 45 1319–1327 15359629 10.1080/1042819042000201989

[B51] Onizuka S. Tawara I. Shimizu J. Sakaguchi S. Fujita T. Nakayama E. (1999). Tumor rejection by in vivo administration of anti-CD25 (interleukin-2 receptor alpha) monoclonal antibody. *Cancer Res.* 59 3128–3133 10397255

[B52] Pobezinsky L. A. Angelov G. S. Tai X. Jeurling S. Van Laethem F. Feigenbaum L. (2012). Clonal deletion and the fate of autoreactive thymocytes that survive negative selection. *Nat. Immunol.* 13 569–578 22544394 10.1038/ni.2292 PMC3362677

[B53] Priatel J. J. Utting O. Teh H. S. (2001). TCR/self-antigen interactions drive double-negative T cell peripheral expansion and differentiation into suppressor cells. *J. Immunol.* 167 6188–6194 11714779 10.4049/jimmunol.167.11.6188

[B54] Punt J. A. Osborne B. A. Takahama Y. Sharrow S. O. Singer A. (1994). Negative selection of CD4^+^CD8^+^ thymocytes by T cell receptor-induced apoptosis requires a costimulatory signal that can be provided by CD28. *J. Exp. Med.* 179 709–713 8294878 10.1084/jem.179.2.709 PMC2191361

[B55] Raiola A. M. Van Lint M. T. Valbonesi M. Lamparelli T. Gualandi F. Occhini D. (2003). Factors predicting response and graft-versus-host disease after donor lymphocyte infusions: a study on 593 infusions. *Bone Marrow Transplant.* 31 687–693 12692609 10.1038/sj.bmt.1703883

[B56] Rieux-Laucat F. Le Deist F. Hivroz C. Roberts I. A. Debatin K. M. Fischer A. (1995). Mutations in Fas associated with human lymphoproliferative syndrome and autoimmunity. *Science* 268 1347–1349 7539157 10.1126/science.7539157

[B57] Riol-Blanco L. Lazarevic V. Awasthi A. Mitsdoerffer M. Wilson B. S. Croxford A. (2010). IL-23 receptor regulates unconventional IL-17-producing T cells that control bacterial infections. *J. Immunol.* 184 1710–1720 20083652 10.4049/jimmunol.0902796 PMC2829977

[B58] Rubocki R. J. Hansen T. H. Lee D. R. (1986). Molecular studies of murine mutant BALB/c-H-2dm2 define a deletion of several class I genes including the entire H-2Ld gene. *Proc. Natl. Acad. Sci. U.S.A.* 83 9606–9610 2879284 10.1073/pnas.83.24.9606 PMC387189

[B59] Sha W. C. Nelson C. A. Newberry R. D. Kranz D. M. Russell J. H. Loh D. Y. (1988). Selective expression of an antigen receptor on CD8-bearing T lymphocytes in transgenic mice. *Nature* 335 271–274 3261843 10.1038/335271a0

[B60] Shimizu J. Yamazaki S. Sakaguchi S. (1999). Induction of tumor immunity by removing CD25^+^CD4^+^ T cells: a common basis between tumor immunity and autoimmunity. *J. Immunol.* 163 5211–5218 10553041

[B61] Shlomchik W. D. (2007). Graft-versus-host disease. *Nat. Rev. Immunol.* 7 340–352 17438575 10.1038/nri2000

[B62] Somasundaram R. Jacob L. Swoboda R. Caputo L. Song H. Basak S. (2002). Inhibition of cytolytic T lymphocyte proliferation by autologous CD4^+^/CD25^+^ regulatory T cells in a colorectal carcinoma patient is mediated by transforming growth factor-beta. *Cancer Res.* 62 5267–5272 12234995

[B63] Strober S. Dejbachsh-Jones S. Van Vlasselaer P. Duwe G. Salimi S. Allison J. P. (1989). Cloned natural suppressor cell lines express the CD3^+^CD4^-^CD8^-^ surface phenotype and the alpha, beta heterodimer of the T cell antigen receptor. *J. Immunol.* 143 1118–1122 2526181

[B64] Su Y. Huang X. Wang S. Min W. P. Yin Z. Jevnikar A. M. (2012). Double negative Treg cells promote nonmyeloablative bone marrow chimerism by inducing T-cell clonal deletion and suppressing NK cell function. *Eur. J. Immunol.* 42 1216–1225 22539294 10.1002/eji.201141808

[B65] Takahashi T. Tanaka M. Brannan C. I. Jenkins N. A. Copeland N. G. Suda T. (1994). Generalized lymphoproliferative disease in mice, caused by a point mutation in the Fas ligand. *Cell* 76 969–976 7511063 10.1016/0092-8674(94)90375-1

[B66] Thomson C. W. Teft W. A. Chen W. Lee B. P. Madrenas J. Zhang L. (2006). FcR gamma presence in TCR complex of double-negative T cells is critical for their regulatory function. *J. Immunol.* 177 2250–2257 16887985 10.4049/jimmunol.177.4.2250

[B67] Tikhonova A. N. Van Laethem F. Hanada K. Lu J. Pobezinsky L. A. Hong C. (2012). αβ T cell receptors that do not undergo major histocompatibility complex-specific thymic selection possess antibody-like recognition specificities. *Immunity* 36 79–91 22209676 10.1016/j.immuni.2011.11.013 PMC3268851

[B68] Van Laethem F. Sarafova S. D. Park J. H. Tai X. Pobezinsky L. Guinter T. I. (2007). Deletion of CD4 and CD8 coreceptors permits generation of alphabetaT cells that recognize antigens independently of the MHC. *Immunity* 27 735–750 18023370 10.1016/j.immuni.2007.10.007

[B69] Voelkl S. Moore T. V. Rehli M. Nishimura M. I. Mackensen A. Fischer K. (2009). Characterization of MHC class-I restricted TCRalphabeta+ CD4- CD8- double negative T cells recognizing the gp100 antigen from a melanoma patient after gp100 vaccination. *Cancer Immunol. Immunother.* 58 709–718 18836718 10.1007/s00262-008-0593-3 PMC2832593

[B70] Watanabe-Fukunaga R. Brannan C. I. Copeland N. G. Jenkins N. A. Nagata S. (1992). Lymphoproliferation disorder in mice explained by defects in Fas antigen that mediates apoptosis. *Nature* 356 314–317 1372394 10.1038/356314a0

[B71] Wong C. K. Ho C. Y. Li E. K. Lam C. W. (2000). Elevation of proinflammatory cytokine (IL-18, IL-17, IL-12) and Th2 cytokine (IL-4) concentrations in patients with systemic lupus erythematosus. *Lupus* 9 589–593 11035433 10.1191/096120300678828703

[B72] Yang L. DuTemple B. Gorczynski R. M. Levy G. Zhang L. (1999). Evidence for epitope spreading and active suppression in skin graft tolerance after donor-specific transfusion. *Transplantation* 67 1404–1410 10385077 10.1097/00007890-199906150-00003

[B73] Yang L. DuTemple B. Khan Q. Zhang L. (1998). Mechanisms of long-term donor-specific allograft survival induced by pretransplant infusion of lymphocytes. *Blood* 91 324–330 9414301

[B74] Ye H. Chang Y. Zhao X. Huang X. (2011). Characterization of CD3^+^CD4^-^CD8^-^ (double negative) T cells reconstitution in patients following hematopoietic stem-cell transplantation. *Transpl. Immunol.* 25 180–186 21911061 10.1016/j.trim.2011.08.004

[B75] Young K. J. DuTemple B. Phillips M. J. Zhang L. (2003a). Inhibition of graft-versus-host disease by double-negative regulatory T cells. *J. Immunol.* 171 134–141 12816991 10.4049/jimmunol.171.1.134

[B76] Young K. J. Kay L. S. Phillips M. J. Zhang L. (2003b). Antitumor activity mediated by double-negative T cells. *Cancer Res.* 63 8014–8021 14633734

[B77] Young K. J. DuTemple B. Zhang Z. Levy G. Zhang L. (2001). CD4(-)CD8(-) regulatory T cells implicated in preventing graft-versus-host and promoting graft-versus-leukemia responses. *Transplant*. Proc. 33 1762–1763 11267502 10.1016/s0041-1345(00)02670-1

[B78] Young K. J. Yang L. Phillips M. J. Zhang L. (2002). Donor-lymphocyte infusion induces transplantation tolerance by activating systemic and graft-infiltrating double-negative regulatory T cells. *Blood* 100 3408–3414 12384444 10.1182/blood-2002-01-0235

[B79] Young K. J. Zhang L. (2002). The nature and mechanisms of DN regulatory T-cell mediated suppression. *Hum. Immunol.* 63 926–934 12368045 10.1016/s0198-8859(02)00446-9

[B80] Zeng D. Liu Y. Sidobre S. Kronenberg M. Strober S. (2003). Activation of natural killer T cells in NZB/W mice induces Th1-type immune responses exacerbating lupus. *J. Clin. Invest.* 112 1211–1222 14561706 10.1172/JCI17165 PMC213484

[B81] Zhang D. Yang W. Degauque N. Tian Y. Mikita A. Zheng X. X. (2007). New differentiation pathway for double-negative regulatory T cells that regulates the magnitude of immune responses. *Blood* 109 4071–4079 17197428 10.1182/blood-2006-10-050625 PMC1874581

[B82] Zhang D. Zhang W. Ng T. W. Wang Y. Liu Q. Gorantla V. (2011a). Adoptive cell therapy using antigen-specific CD4^-^CD8^-^ T regulatory cells to prevent autoimmune diabetes and promote islet allograft survival in NOD mice. *Diabetologia* 54 2082–2092 21594554 10.1007/s00125-011-2179-4

[B83] Zhang Z. X. Lian D. Huang X. Wang S. Sun H. Liu W. (2011b). Adoptive transfer of DNT cells induces long-term cardiac allograft survival and augments recipient CD4(+)Foxp3(+) Treg cell accumulation. *Transpl. Immunol.* 24 119–126 21073952 10.1016/j.trim.2010.11.003

[B84] Zhang Z. X. Ma Y. Wang H. Arp J. Jiang J. Huang X. (2006). Double-negative T cells, activated by xenoantigen, lyse autologous B and T cells using a perforin/granzyme-dependent, fas–fas ligand-independent pathway. *J. Immunol.* 177 6920–6929 17082607 10.4049/jimmunol.177.10.6920

[B85] Zhang Z. X. Stanford W. L. Zhang L. (2002). Ly-6A is critical for the function of double negative regulatory T cells. *Eur. J. Immunol.* 32 1584–1592 12115641 10.1002/1521-4141(200206)32:6<1584::AID-IMMU1584>3.0.CO;2-2

[B86] Zhang Z. X. Yang L. Young K. J. Dutemple B. Zhang L. (2000). Identification of a previously unknown antigen-specific regulatory T cell and its mechanism of suppression. Nat Med 6 782–789 10888927 10.1038/77513

